# Novel N4-Like Bacteriophages of *Pectobacterium atrosepticum*

**DOI:** 10.3390/ph11020045

**Published:** 2018-05-14

**Authors:** Colin Buttimer, Hanne Hendrix, Alan Lucid, Horst Neve, Jean-Paul Noben, Charles Franz, Jim O’Mahony, Rob Lavigne, Aidan Coffey

**Affiliations:** 1 Department of Biological Sciences, Cork Institute of Technology, T12 P928 Cork, Ireland; colin.buttimer@mycit.ie (C.B.); alanlucid@gmail.com (A.L.); Jim.OMahony@cit.ie (J.O.); 2 Laboratory of Gene Technology, KU Leuven, 3001 Leuven, Belgium; hanne.hendrix@kuleuven.be (H.H.); rob.lavigne@kuleuven.be (R.L.); 3 Department of Microbiology and Biotechnology, Max Rubner-Institut, 24103 Kiel, Germany; horst.neve@mri.bund.de (H.N.); charles.franz@mri.bund.de (C.F.); 4 Biomedical Research Institute and Transnational University Limburg, Hasselt University, 3590 Hasselt, Belgium; jeanpaul.noben@uhasselt.be; 5 APC Microbiome Institute, University College, T12 YT20 Cork, Ireland

**Keywords:** phage isolation, phage characterization, *Pectobacterium*, blackleg, soft rot, phage-mediated biocontrol of bacteria, plant disease biocontrol, phage therapy, N4-like phage

## Abstract

*Pectobacterium atrosepticum* is an economically important phytopathogen that is responsible for potato blackleg and soft rot, and for which current control strategies are limited. In this study, stem samples of potato crops exhibiting blackleg were taken from three farms in Co. Cork, Ireland, and they were found to be infected with *P. atrosepticum*. Three closely related bacteriophages (phages) that are specific to this phytopathogen were isolated and characterized, namely vB_PatP_CB1, vB_PatP_CB3, and vB_PatP_CB4 (abbreviated as CB1, CB3, and CB4). Both CB1 and CB3 were determined to infect 12 strains and CB4 10 strains of the 19 strains of *P. atrosepticum* tested. Morphology, latent periods, burst sizes, and their stability at various temperatures and pHs were also examined. Genome sequencing of the three phages revealed that they shared a minimum nucleotide identity of 93% with each other. Their genomes exhibited an *Enquartavirinae* genome organization, possessing several conserved proteins that were associated with phages of this group, like the type species *Escherichia* virus N4. Tandem electrospray ionization-mass spectrometry (ESI-MS/MS) allowed for the identification of ten structural proteins that form the virion of CB1, six that are conserved in phage N4. Biocontrol experiments demonstrated that the phages suppress soft rot formation upon co-inoculation with *P. atrosepticum* on whole tubers. The results of this study indicate that CB1 related phages could be good candidates for phage-based control.

## 1. Introduction

The genera of *Pectobacterium* and *Dickeya*, which are often referred to collectively as the soft rot *Enterobacteriaceae* (SRE), are phytopathogens that cause economically important losses in a wide range of arable and ornamental crops. These bacteria are all Gram-negative, non-spore forming, facultative anaerobic rods that are typified by the production of extracellular pectinolytic enzymes during plant infection [1,2]. Both genera are considered to be among the top ten most important plant pathogens [3].

To date, the predominant SRE species and subspecies that cause blackleg and soft rot of the potato crop in Europe are *Pectobacterium atrosepticum*, *Pectobacterium carotovorum* subsp. *carotovorum*, *Pectobacterium parmentieri* (formally *Pectobacterium wasabiae*), *Dickeya dianthicola*, and *Dickeya solani* [1,4,5,6]. *P. atrosepticum* is traditionally believed to be one of the more important causative agents for blackleg in temperate climates [7], and the dominant blackleg causative agent in Scotland [5].

Strategies for the control of potato soft rot and blackleg are limited, whether they be physical, chemical, or biological. In addition, no blackleg resistant potato cultivars are available. The current approach to disease control is the use of cultivation practices to minimize levels of infection, including contamination avoidance and the removal of diseased plants and/or tissue. Seed certification schemes are also employed. However, the success of these schemes vary and are highly weather dependent [8,9].

Phages, which are the viruses of bacteria, are being investigated as a potential biocontrol strategy for many problematic bacteria, including the phytopathogens. Indeed, phages of *Erwinia amylovora*, *Xanthomonas campestris* pv. *vesicatoria*, *Agrobacterium tumefaciens*, and many others have been investigated for their disease control potential [10,11]. In addition, a number of SRE phages have also been recently isolated and characterized [12], some of these having also been assessed for their potential as biocontrol agents of their respective phytopathogenic hosts, with promising results.

Proof of concept experiments using SRE phages on potato whole tubers have demonstrated that phage biocontrol has the capability to inhibit soft rot caused by *P. carotovorum* subsp. *carotovorum*, *P. parmentieri*, and *D. solani* [13,14,15]. A field trial was conducted by Adriaenssens et al. [14] using phage vB_DsoM_LIMEstone1 against *D. solani* and showed that phage treatment against blackleg resulted in decreased disease severity and improved yields. However, SRE species causing disease that were not sensitive to the phage limited the success of the overall outcome. Investigation of phage biocontrol of SRE also has not been limited to the potato. Phage biocontrol was shown, for example, to reduce the incidence of soft rot by 50% on tuber plugs of Calla lily due to *P. carotovorum* subsp. *carotovorum* infection in greenhouse trials [16]. Promising results were also demonstrated using phage against this species infecting Chinese cabbage [17].

We here report the isolation and characterization of *P. atrosepticum* phages vB_PatP_CB1, vB_PatP_CB3, and vB_PatP_CB4. To the authors’ knowledge, this is the first report of N4-like phages to be identified infecting bacteria belonging to SRE. *Escherichia* phage N4 is a lytic podovirus, which was isolated in the 1960s from sewers in Genoa, Italy [18]. The phage is typified by the possession of a virion-associated RNA (vRNA) polymerase, which it injects along with its genome into its host *Escherichia coli* at the beginning of infection to initiate transcription of its DNA and an overall conserved transcriptional scheme [19]. To date, Genbank contains at least 56 genome sequences of N4-like phages infecting hosts belonging to the classes of *Alpha-*, *Beta-*, and *Gamma-proteobacteria*, with each phage genome encoding the hallmark feature of a vRNA polymerase. Six phage genera have been defined among these so far, these being *G7cvirus*, *Lit1virus*, *Ea92virus*, *Luz7virus*, and *N4virus* [20,21,22,23] with another two genera (*Sp58virus* and *Dss3virus*) having been proposed [21]. Furthermore, using these newly isolated N4-like phages, we demonstrated their potential for biocontrol with the suppression of soft rot formation with their co-inoculation with *P. atrosepticum* on potato tubers.

## 2. Results

### 2.1. Isolation of SRE from Potato Crops Symptomatic of Blackleg

In 2013, nineteen potato stem samples symptomatic for blackleg, each representing a crop, were collected from three distinct farms in Co. Cork, Ireland. Isolate identification was based on cavity formation on crystal violet pectate (CVP) medium, production of reducing substances from sucrose, acid production from α-methyl-glucoside, *Pectobacterium* genus-specific along with *P. atrosepticum* and *P. carotovorum* subsp. *carotovorum* species-specific polymerase chain reactions (PCRs), and matrix assisted laser desorption/ionization-time of flight (MALDI-TOF) mass spectrometry. Based on these results, fourteen plants were found to be infected with *P. atrosepticum* with a remaining plant being found to be infected with both *P. atrosepticum* and *P. carotovorum* subsp. *carotovorum* (Supplementary Information 1, Table S1).

### 2.2. Isolation of Phages, Host Range and General Characteristics

**Figure 1:**
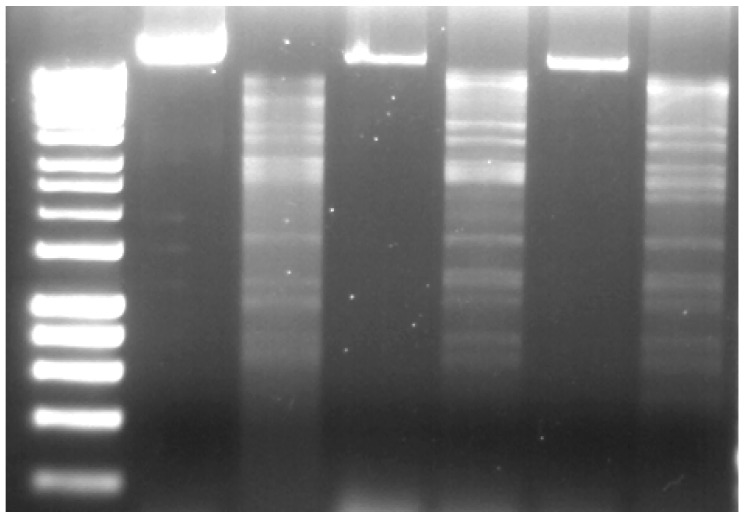
Genomic DNA of *Pectobacterium* phages CB1, CB3, and CB4, BamHI-digested (lanes 3, 5, and 7, respectively) and undigested (lanes 2, 4, and 6, respectively). Lane 1, DNA marker (Hyperladder 1 kb, Bioline). Gel concentration 1% *w*/*v* agarose.

Thirteen phage isolates were obtained from soil samples that were collected from potato grading machinery and potato fields from two of the three farms that are mentioned above. These were subjected to genomic DNA comparison. Restriction digestion analysis, employing BamHI, allowed for the identification of ten isolates that produced three similar band patterns (Figure 1). Phages producing these patterns were found in both the grading machinery and the field soils. An example of each was taken for further study, namely CB1, CB3, and CB4. These isolates produced clear plaques with an approximate diameter of 2–3 mm (overlay concentration 0.4% *w*/*v* agar in LB) on their respective host strains of *P. atrosepticum*, with narrow halos occasionally being observed to the surrounding plaques.

The host ranges of these phages were examined using 31 bacterial strains (local Irish isolates and reference strains) from five different species belonging to SRE, namely *P. atrosepticum* (19 strains), *P. carotovorum* subsp. *carotovorum* (four strains), *D. chrysanthemi* bv. *chrysanthemi* (one strain), *D. dianthicola* (three strains), and *D. solani* (four strains). These phages were able to form plaques on 15 strains of their host species, *P. atrosepticum*, with no plaque formation or inhibition of growth being observed on strains of the other species. Slight variations in lytic ability were found among the three phages, with CB1 and CB3 determined to form plaques on 12 strains, while CB4 was only found to infect 10 of the 19 strains that were tested (Table 1).

**Figure 2:**
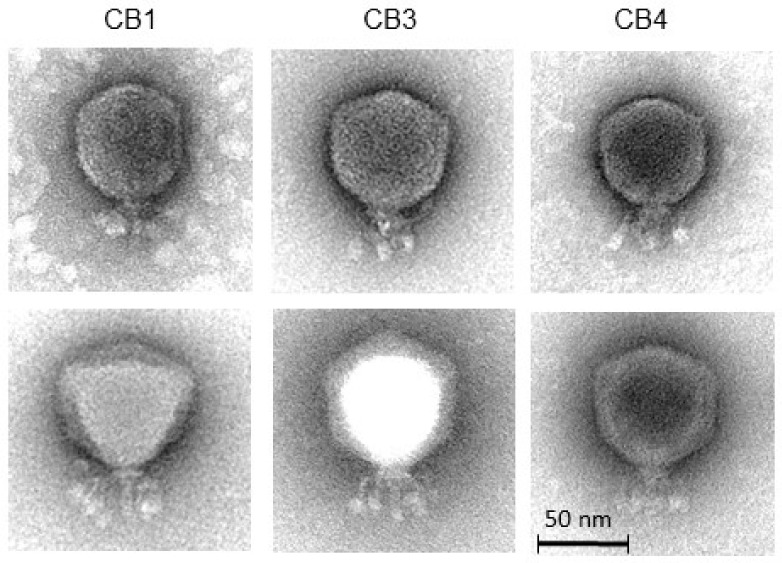
Transmission electron micrographs of negatively stained *Pectobacterium* phages CB1, CB3, and CB4 using 2% (*w*/*v*) uranyl acetate (**top**) and 1% (*w*/*v*) phosphotungstic acid (**bottom**).

Examination of the morphology of the three phages by transmission electron microscopy showed that they can be classified as members of the *Podoviridae* family [24]. They featured a C1 morphotype with isometric capsids (ca. 70 nm) and short non-contractile tails (length: ca. 25 nm) (Figure 2 and Table 2). Head and tail measurements are consistent with previously reported N4-like phages [21]. Additionally, a set of (putatively six) short whiskers (length: ca. 25 nm) that were attached to a collar structure (width: ca. 19 nm) were observed. At their distal ends, the whiskers terminate with elongated globular appendices (ca. 12 nm × 7 nm). The three phages were named in accordance with the nomenclature set out by Kropinski et al. [25].

The one-step-growth curve assay, under standard conditions using LB medium, showed that the latent period of CB1 was 60 min with an approximate burst size of 207 plaque forming units (PFU)/cell. For CB3, a latent period of 65 min with an approximate burst size of 246 PFU/cell was observed. For CB4, a latent period of 65 min with an approximate burst size of 158 PFU/cell observed (Supplementary Information 1, Figure S1). Phage viability under different environmental conditions was also examined. Over a duration of one hour, all of the phages were found to be stable between −18 °C and 50 °C. They were also found to be stable between pH 5 and 11 for 24 h (Supplementary Information 1, Figures S2 and S3).

### 2.3. Genome and Proteome Analysis

#### 2.3.1. Genomes of Phages CB1, CB3, and CB4 Show an N4virus Organization

The genome sequences obtained for phages CB1, CB3, and CB4 were 75,394 bp, 75,522 bp, and 75,973 bp with coverage of 2057×, 1882×, and 1765×, respectively. The genomes of these phages are likely linear with direct terminal repeats (DTRs) with estimated sizes of 647 bp (for CB1 and CB3) and 648 bp (for CB4). This estimation is based on the identification of a localized region with roughly double the read depth in comparison to average read depth across the whole genome of each phage, a similar finding to the previously reported N4-like phages [26,27]. However, it is noteworthy that termini at the ends of the DTRs for these phages can be asymmetric, as seen in the case of *Escherichia* phage N4 itself [28]. It is not known whether DTR asymmetry exists in phages CB1, CB3, and CB4. As well as this, the average GC content of these genomes was found to be 49%, which was just below the GC content associated with the host bacterium *P. atrosepticum* at 50–51% [29,30].

**Figure 3:**
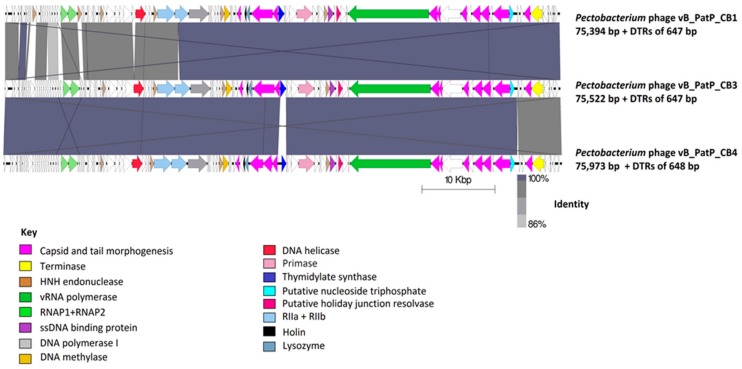
Comparison of the genomes of *Pectobacterium* phages CB1, CB3, and CB4 employing BLASTN and visualized with Easyfig. Genome maps comprise of arrows indicating the locations of open reading frames (ORFs) among the different phage genomes. Arrows have been color-coded describing their predicted roles (see key), and lines between genome maps indicate levels of homology.

The number of predicted open reading frames (ORFs) that is determined on the non-redundant genome of these phages were 97, 102 and 100 for CB1, CB3, and CB4, respectively (Supplementary Information 2–4). Proteins that were predicted to play roles in transcription, DNA replication, virion morphogenesis, and host lysis were identified in all three phages as well as putative genes for homing endonucleases (Figure 3). No integrase, excisionase, or repressor genes were detected, suggesting that all three phages have an exclusively lytic lifecycle. Moreover, no ORFs were identified for pathogenicity or known toxins.

Phages CB3 and CB4 exhibited a 97% genome-wide nucleotide identity (BLASTN algorithm) and can thus be considered different isolates of the same phage species [31]. The major feature variations found between them being their ORFs for thymidylate synthase and N4 gp32-like protein. The only major difference identified with a host range between these phages was the ability of CB3 to infect two extra *P. atrosepticum* strains (CB BL5-1 and CB BL18-1) (Table 1). In addition, phage CB4 also possesses two genes for tRNAs (for asparagine & glutamine). In contrast, phage CB1 was only 95% similar to CB3 and 93% similar to CB4. This places CB1 on the boundary of speciation with the other two phages. CB1 possesses the same predicted thymidylate synthase gene and it has no tRNA genes like CB3. However, the predicted early gene region possesses variations in its ORF content not shared with CB3 and CB4. These differences include the presence of different hypothetical proteins and the presence of two additional HNH endonucleases. There is also notable sequence variation of the rIIB protein of CB1 to that of the other two phages. Interestingly, CB1 shares its N4 gp32-like gene with CB4 (Supplementary Information 1, Table S2).

**Figure 4:**
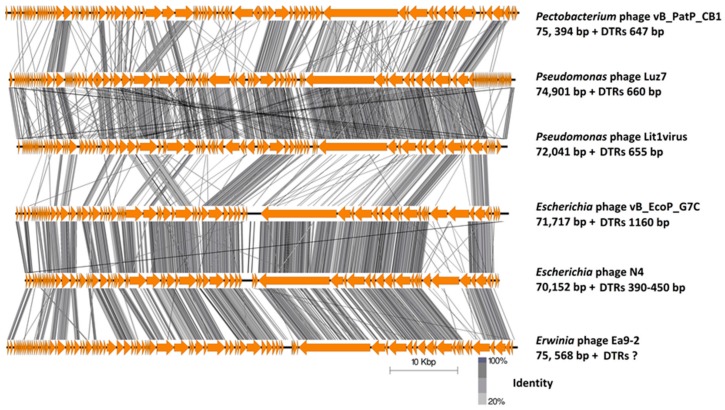
Comparison of the genomes of *Pectobacterium* phage CB1 with those phages representing the genera of *N4virus*, *G7cvirus*, *Ea92virus*, *Lit1virus*, and *Luz7virus* employing TBLASTX and visualized with Easyfig. Genome maps comprise of arrows indicating the locations of genes on the different phage genomes, and lines between genome maps indicate the level of homology.

**Figure 5:**
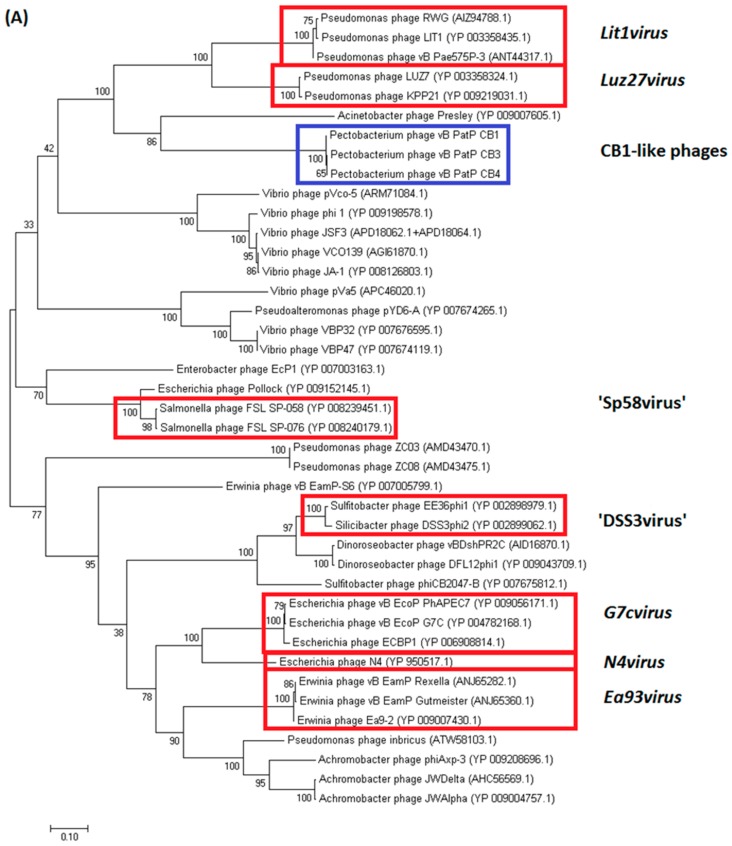

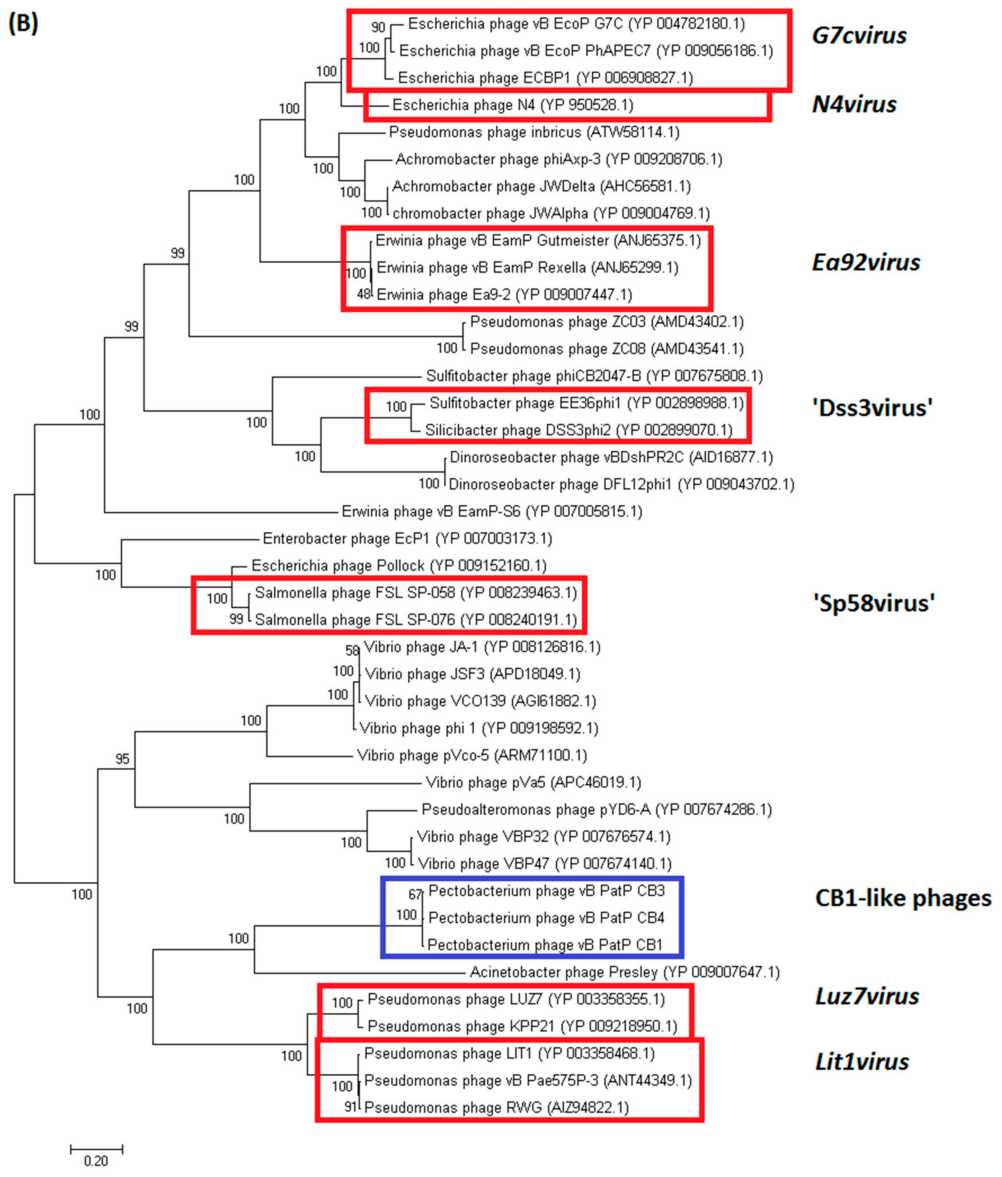
Phylogenetic analysis using the (**A**) DNA polymerase (log likelihood = −16,535.57) and (**B**) vRNA polymerase (log likelihood = −103,761.67) protein sequences of *Pectobacterium* phages CB1, CB3 and CB4 and 38 other N4-like phages. Phages belonging to the genera of *G7cvirus*, *Lit1virus*, *Ea92virus*, *Luz7virus*, and *N4virus* and proposed genera *Sp58virus* and *Dss3virus* are highlighted. The amino acid sequences were compared using MUSCLE. The tree was constructed using the maximum likelihood algorithm. The percentages of replicate trees were assessed with the bootstrap test (1000).

**Figure 6:**
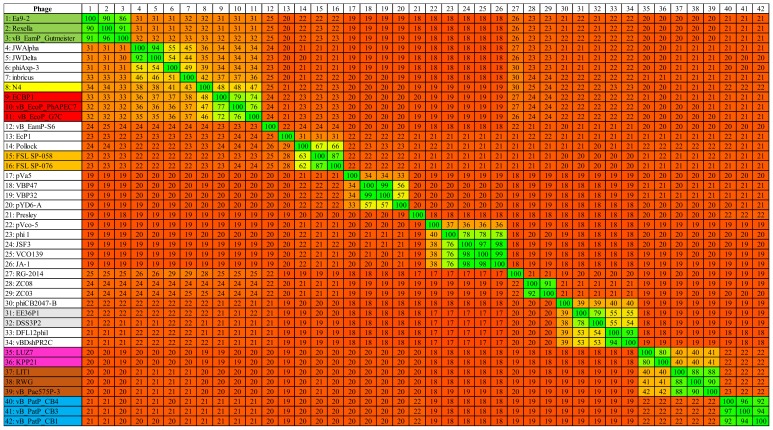
TBLASTX heat map generated using Gegenees with accurate parameters – fragment length: 200 bp; and step size: 100 bp; threshold: 0%. The map includes the genomes of 38 N4-like phages with phages representing the genera *G7cvirus* (red), *Lit1virus* (brown), *Ea92virus* (green), *Luz7virus* (pink), and *N4virus* (yellow) and proposed genera *Sp58virus* (orange) and *Dss3virus* (grey), with the *Pectobacterium* phages CB1, CB3 and CB4 coded in blue.

The overall genomic architecture of the CB1-like phages resembles those that are belonging to the N4-like group (Figure 4), possessing all 18 core proteins of these phages that were identified by Li et al. [32] (Supplementary Information 1, Table S3), thus allowing the N4-like designation. When comparing these phages to *Escherichia* phage N4, the gene order of most structural proteins is well conserved with major variations associated with the genes that are involved in host lysis and the genes for structural proteins believed to play roles in tail morphogenesis. This observation has been noted among other N4-like phages [33,34]. These phages do not fall within any genera currently defined in the N4-like group to date. Phylograms that are based on the vRNA and DNA polymerase proteins show that they form their own distinct clade among these phages (Figure 5), with their closest evolutionary relationship appearing to be with the phages of the genera of *Lit1virus* and *Luz7virus*. Additionally, when protein homology was calculated with Gegenees analysis (TBLASTX), they show limited identity to other N4-like members (Figure 6).

#### 2.3.2. Transcription

Transcription of the CB1-like phage genomes likely happens in a similar manner to that of *Escherichia* phage N4. All three phages possess a vRNA (CB1_77, CB3_82, CB4_81), which conducts the transcription of single-stranded DNA that is initiated at hairpin promoters that are located within the early gene region. These promoters are composed of a five-nucleotide hairpin with a three-nucleotide loop possessing a central purine [19]. Inspection of the early gene region of phages CB1, CB3, and CB4 reveal the presence of three potential hairpin loops for all three, which appear analogous to hairpin loops in phage N4 (Supplementary Information 1, Table S4). Middle gene transcription in phage N4 is conducted by a heterodimeric RNA polymerase that is related to the T7-like RNA polymerase family [35], with the CB1-like phages also possessing homologs (CB1_22, 23, CB3_24, 25, and CB4_24, 25). Finally, late gene transcription of phage N4 involves its ssDNA-binding protein activating the *E. coli* sigma 70 RNA polymerase directing it towards the N4 late promoters [36], with the ssDNA-binding protein also having homologs among the CB1-like phages (CB1_72, CB3_77, and CB4_76). Within the non-redundant parts of the genomes of the CB1-like phages, 18 putative rho-independent terminators were identified for CB1 and CB3, with 17 being identified for CB4 (Supplementary Information 1, Tables S5–S7). Comparison between these phages showed that 16 of these terminators were located at similar locations among their respective genomes (Supplementary Information 1, Table S8).

#### 2.3.3. DNA Replication, Metabolism and Methylation

The CB1-like phages have several components of a DNA replication system, including a DNA polymerase I (CB1_46, CB3_50, CB4_49), a helicase (CB1_40, CB3_42, CB4_42), and a primase (CB1_67, CB3_72, CB4_71). Their ability to alter the nucleotide pool of their host exploiting a thymidylate synthase (CB1_63, CB3_68, CB4_67) allows for the conversion of dUMP to dTMP and a putative nucleoside triphosphate pyrophosphohydrolase (CB1_87, CB3_92, CB4_91), which allows for the conversion of nucleoside triphosphates to their monomer form. These phages also possess a DNA adenine methylase (CB1_53, CB3_57, CB4_56) (IPR012327). Indeed, restriction digestion patterns of the genomic DNA of CB1, CB3 and CB4 using ClaI indicate that their DNA is likely deoxyadenosine-methylated (Supplementary Information 1, Figures S4–S6). Such methylation is known to occur on the DNA of a number of other phages, such as P1 and T4 of *E. coli*. It can have regulatory functions that are involved in phage DNA packaging and transcription, but additionally, can provide resistance against host restriction endonucleases [37].

#### 2.3.4. Cell Lysis

*Escherichia* phage N4 possesses a signal-anchor-release (SAR) endolysin (*N*-acetylmuramidase, pfam05838) [38]. Such endolysins are transported to the inner membrane by the host *sec* system and they depend on pin holins to cause the collapse of membrane potential to induce their cell wall degrading activity [39]. A typical feature of such endolysins is the possession of an N-terminal transmembrane domain. However, the CB1-like phage endolysin (Endolysin lambda type, IPR034691) (CB1_60, CB3_64, CB4_63) lacks this feature, thus indicating that their endolysin likely depends on the predicted class II holin (CB1_59, CB3_63, CB4_62) for release into the host cell periplasm to reach cell wall peptidoglycan [39]. Situated next to these predicted ORFs for the endolysin and holin of the CB1-like phages are two overlapping ORFs (CB1_58, 58a, CB3_62, 62a, CB4_61, 61a), which possess one of the typical gene arrangements of a spanin rz and rz1 pair [40]. In this spanin pair arrangement, the gene for the rz protein is the larger of the two, encoding a protein with a transmembrane domain. The smaller gene encodes a lipoprotein. However, in the CB1-like example, the ORF that is predicted to encode the lipoprotein is the larger of the two with the smaller protein lacking the predicted transmembrane domain.

#### 2.3.5. Structural Proteome of Phage CB1

There is a minimum of ten proteins that have been identified to form the virion of *Escherichia* phage N4 [41], with *in silico* analysis showing six to be shared with phage CB1. These six are the vRNA polymerase (CB1_77), the major capsid (CB1_83), the portal protein (CB1_86), and structural proteins of unknown function resembling those of gp52 (CB1_79), gp54 (CB1_81), and gp67 (CB1_90) of N4. Those that were not found to be shared with CB1 were the tail sheath (gp65), the tail appendage (gp66), the head decorating protein (gp17), and one other structural protein (gp51) that has previously been suggested to be an internal virion protein [42]. Using *in silico* analysis, three proteins were identified to play potential roles in the morphogenesis of the tail structure of CB1 not shared with N4, namely CB1_57, 61, and 62. The CB1_61 gene product is likely a tail spike given that it possesses an SGNH hydrolase-type esterase domain (IPR013830), suggesting enzymatic activity, like the tail spike (gp63.1) of N4-like *Escherichia* phage G7C, which deacetylates host surface polysaccharides [43]. Mass spectrometry (ESI-MS/MS) that was conducted on the structural proteome of CB1 verified the presence of the shared N4 structural proteins along with the three identified putative tail proteins and one hypothetical protein (CB1_78) (Table 3 and Supplementary Information 5, Table S1). This latter protein of the CB1 virion mirrors the position of gp51 of phage N4, but shares no homology. All of the CB1 structural proteins that were identified possess homologs in phages CB3 and CB4, with the putative tail spike protein CB1_61 being split into two ORFs on the genome of CB4 (CB4_64, 65). Furthermore, the large terminase responsible for virion DNA packaging was identified for all three phages (CB1_91, CB3_97, CB4_95).

#### 2.3.6. Selfish Genetic Elements

Homing endonucleases are mobile genetic elements with endonuclease activity that only promote the spread of their own encoding gene [41]. Several homing endonucleases of the HNH family (IPR003615) have been identified on the genomes of the three CB1-like phages. Phage CB1 itself was found to possess six homing endonucleases (CB1_24, 33, 39, 43, 52, 71), whereas phages CB3 (CB3_36, 47, 56, 76) and CB4 (CB4_36, 46, 55, 75) both possess four. Such genes have also been identified among other N4-like phages [26,34].

### 2.4. Phage Biocontrol on Whole Tubers

The phage mixture consisting of phages CB1, CB3, and CB4 was assessed for its ability to suppress soft rot (in a whole tuber assay) caused by a mixed infection by their host *P. atrosepticum* strains DSM 18077 and DSM 30186. The potato cultivar Rooster was selected for these experiments, given that it is the predominant variety that is grown in the Republic of Ireland (it comprised 60% of the Irish crop in 2014) [44].

**Figure 7:**
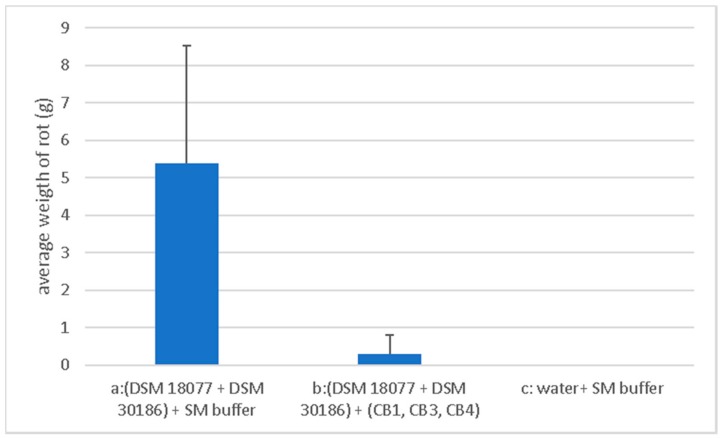
Protective effective effect of a phage mixture containing *Pectobacterium* phages CB1, CB3, and CB4 on whole tubers against a mixed infection of *Pectobacterium atrosepticum* strains DSM 18077 and DSM 30186. The assay (n = 10) was carried out independently in triplicate and results were averaged.

The whole tuber rot assay was carried out independently in triplicate, and the results were averaged. Assay involved two sets of ten tubers that were treated with bacteria (designated sets (a) and (b), 100 µL at approx. 1.0 × 10^7^ CFU/mL), which were allowed to absorb into the tuber tissue. A third set (c) was treated with water. Sets (a) and (c) were treated with SM, while (b) was treated with the phage mixture (100 µL at 1 × 10^7^ PFU/mL). Following incubation, the average weight of rotten tissue from each set of tubers was recorded. Set (a) (bacteria + SM buffer) was 5.39 ± 3.121 g; Set (b) (bacteria + phage) was 0.311 ± 0.498 g, thus the average weight of infected tubers that were treated with phage was less than that treated without phage, with this result being statistically significant (*p* < 0.0005). Therefore, indicating that phage treatment limited soft rot formation. No rot was observed for set (c) (Figure 7). Look to Supplementary Information 1, Table S7 for the visual outcome of treated tuber sets (a), (b) and (c).

## 3. Discussion

Of the sixteen samples of potato crops symptomatic of blackleg that were obtained from three potato farms in Co. Cork, from which pectolytic isolates were found, fifteen were identified to be infected with *P. atrosepticum* with the remaining infection being identified to be caused by a mixture of *P. atrosepticum* with *P. carotovorum* subsp. *carotovorum.* This study focused on the identification and isolation of exclusively lytic phages of *P. atrosepticum* from the potato crop environment, followed by an assessment of their potential for biocontrol applications against soft rot and/or blackleg disease. *P. atrosepticum* is highly relevant to Irish potato horticulture, and to date, relatively few reports focusing on phage biocontrol of this pathogen have appeared in the scientific literature.

During an enrichment screening for *P. atrosepticum* phages, three closely related phage isolates were obtained, namely CB1, CB3, and CB4. Genome sequencing revealed them to be N4-like, sharing a similar gene order and possessing all 18 core genes that were found among all N4-like members. For phage therapy applications involving the treatment of *Pseudomonas aeruginosa* murine infections, N4-like phages have been deemed to be safe and effective [45]. This suggested that the three identified phages could be good candidates for biocontrol applications.

Phages CB1, CB3, and CB4 appear to possess relatively broad host ranges within their host species, collectively spanning 15 of the 19 tested *P. atrosepticum* strains (Table 1). By comparison, among the other members of the N4-like phages (infecting *Escherichia*, *Pseudomonas*, *Vibrio*, and *Roseovarius*), many have been found to propagate only on the strain used in the original isolation [34,46,47]. The relative broad host range that was observed is desirable for their use in biocontrol applications. Nevertheless, the application of these phages would have limited biocontrol potential where other SRE species may be involved in disease. Additionally, *P. atrosepticum* strains that were tested consisted predominantly of those from an Irish environment. It is unknown if strains from a different geographical location would have a similar susceptibility to these phages. However, resistance could be overcome by supplementing the phage mixture with additional phages targeting resistant *P. atrosepticum* strains, as well as other SRE species. The generation of phage cocktails is an important approach being adopted for phage preparations for use in the food industry and veterinary medicine as it ensures the widest possible host range against the targeted bacteria [48,49]. Phages with host ranges that were limited within their respective host species have been observed for a number of other studies focusing on SRE phage biocontrol [13,14,17]. Nevertheless, a few notable exceptions have been described, such as *Dickeya* phages D4 and D5 being able to infect several *Dickeya* species and *Dickeya* phages ϕPD10.3 and ϕPD23.1 being able to infect *D. solani*, *P. carotovorum* subsp. *carotovorum* and *P. parmentieri* [12,13]. To date, no broad host range phage infecting multiple SRE species, including *P. atrosepticum*, has been described.

The tuber rot assay in this study indicated that phages CB1, CB3, and CB4 appeared to possess some potential for the inhibition of soft rot formation of potato tubers (Figure 7). A similar finding was previously demonstrated using phages against *P. carotovorum* subsp. *carotovorum*, *D. solani*, and *P. parmentieri* [13,14,15]. An approximate multiplicity of infection (MOI) of 1 was used in our experiments to achieve this effect, lower than that reported with *Dickeya* phage vB_DsoM_LIMEstone1 at a MOI of 10 and 100 [14], but higher than that with *Dickeya* phages ΦPD10.3 and ΦPD23.1 against *P. carotovorum* subsp. *carotovorum*, *D. solani*, and *P. parmentieri* at an MOI of 0.01 [15]. However, further work is necessary to examine the inhibitory effect of soft rot on potato tubers by the CB1-like phages in more detail as well as additional studies to explore if the phages could inhibit blackleg formation on whole plants. Obviously, other environmental conditions that could potentially affect the viability of these phages in field applications, such as UV light, salinity, soil composition, and agrochemical factors (including fertilizers etc.) would also require investigation.

Of the ten structural proteins that were identified to form the CB1 virion (Table 3), four were not shared with that of *Escherichia* phage N4 (CB1_57, 61, 62, and 78). These proteins appear to be associated with tail components of the CB1 virion. Their lack of homology is likely due to their adaption to allow for the recognition of its host bacterium and enable subsequent DNA injection. For N4-like *Escherichia* phage G7C to recognize its host, its tail spike esterase domain must first deacetylate the O-antigen of its host lipopolysaccharide [43]. This may also be the case for the CB1-like phages with their putative tail spike (CB1_61, CB3_66, and CB4_75, 76) possessing an SGNH hydrolase-type esterase domain (IPR013830).

Phylogenetic analysis (based on phylograms and Gegenees (TBLASTX) of CB1-like phages show that they are distinct members within the N4-like group, with their closest evolutionary relationship being found with phages of the genera *Lit1virus* and *Luz7virus*. This analysis also indicates the existence of higher order taxonomic relationships between genera of the N4-like phages. Such relationships are becoming apparent due to the increasing number of N4-like genomes being added to public databases. Such a relationship was identified between the phages belonging to the genera of *N4virus*, *G7cvirus*, and *Ea92virus* with *Achromobact*er phage JWAlpha, which has been proposed to form the subfamily *Enquartavirinae* [21]. A similar connection was also found with the genera of *Luz7virus* and *Lit1virus* (Figure 5 and Figure 6). These relationships are indicated with clades that are closely situated within phylograms and the sharing of protein sequence identity of ~30–40% in Gegenees analysis.

## 4. Materials and Methods

### 4.1. Bacterial Strains, Phage and Cultivation Conditions

CVP agar was used to isolate *Pectobacterium* strains from stems of potato plants presenting symptoms of blackleg [50]. Bacterial identification was achieved by using biochemical and physiological tests [51], genus and species-specific PCRs [52,53,54], and MALDI-TOF mass spectrometry (Bruker Daltonics Biotyper, Bruker, Billerica, MA, USA). To grow bacterial strains and to propagate phage Lysogeny broth (LB), LB agar (1.5% *w*/*v* agar) and LB overlays (0.4% *w*/*v* agar) were used. All cultures were grown at 25 °C unless stated otherwise. Phages were propagated on *P. atrosepticum* strains DSM 18077 (for phage CB1) and DSM 30186 (for phages CB3 and CB4).

### 4.2. Phage Isolation

Phages were isolated using an enrichment method. Five grams of soil was weighed out and were placed into 30 mL of LB broth, along with the addition of 300 µL of an overnight culture of *P. atrosepticum* (DSM 18077, DSM 30184, DSM 30185, and DSM 30186) and then incubated for 18 h at 25 °C. This was centrifuged to pellet soil matter with supernatant then being filtered (0.45 µm pore-size filter, Sarstedt, Nümbrecht, Germany). The supernatant was spotted (10 µL) onto LB overlays that were seeded with different strains of *Pectobacterium*. Phages were isolated by picking individual plaques and then replating and reisolating to ensure purity [55].

### 4.3. Host Range and General Characterization

Host range of phages was tested by spotting a serial dilution (neat to 10^−7^) of a phage suspension (titer of 10^7^ PFU/mL) onto LB overlays that were seeded with bacteria of interest. EOP values were determined for sensitive strains by dividing phage titer on target bacterium by phage titer on host bacterium. Bacterial strains used in host range study are listed in Supplementary Information 1, Table S1, and Supplementary Information 6, Table S1.

One-step-growth curve assay was performed in a similar manner as previously described [56,57]. Phage host strains were grown to an OD600 of 0.20–0.23 (approx. 1 × 10^8^ CFU/mL), followed by centrifugation of 2 mL in a microfuge to pellet bacteria. The pellet was resuspended in 1 mL of phage suspension to yield an approx. MOI of 5 × 10^−4^ following incubation at 25 °C for one minute. This was then centrifuged to pellet bacteria and the supernatant was removed, thus separating bound from unbound phages. The bacterial pellet with bound phage was then resuspended in 10 mL of LB and incubated aerobically in a water bath at 25 °C with agitation at 60 rpm. At five-minute intervals, aliquots were removed to measure phage titer by the overlay method. Based on the number of PFU/mL, the latent period and the burst size were determined, by dividing the average PFU/mL of the latent period by the average PFU/mL of the last four time points of the experiment.

Phage stability was tested by incubating phage suspension of 10^6^ PFU/mL in SM buffer (50 mM Tris.HCl pH 7.5, 100 mM NaCl, 8 mM MgSO_4_) at different temperatures for one hour and incubating phage suspension in pH buffer ranging from 2 to 12 (10 mM trisodium citrate, 10 mM boric acid, and 150 mM KCl, adjusted with NaOH or HCl) for 24 h [14].

### 4.4. CsCl Gradient Purification

Isopycnic centrifugation through CsCl gradients was performed, as previously described [58], with a number of modifications. A high titer phage lysate (>1 × 10^9^ PFU/mL), was precipitated using polyethylene glycol (15% *w*/*v* PEG8000, 1 M NaCl) at 4 °C overnight and centrifuged, after which the pellet was resuspended in TMN buffer (10 mM Tris-HCl pH 7.4, 10 mM MgSO_4_·7H_2_O, 0.5 M NaCl), and where necessary a chloroform phase separation step (1:1) was conducted to remove debris. The resulting phage preparation was placed onto a CsCl step gradient composed of 1.3, 1.5, and 1.7 g/mL layers and spun in a 100 Ti rotor (Beckman Coulter, Brea, CA, USA) at 200,480 g for 3 h at 4 °C. Resulting phage bands were collected and subjected to dialysis with two changes of Tris-HCl buffer (10 mM, pH 7.5) at 4 °C.

### 4.5. Transmission Electron Microscopy

Phages were negatively stained on freshly prepared ultra-thin carbon films with 2% (*w*/*v*) uranyl acetate and with 1% phosphotungstic acid, as described in detail earlier [59]. Micrographs were taken using a Tecnai 10 transmission electron microscope (FEI Thermo Fisher, Eindhoven, The Netherlands) at an acceleration voltage of 80 kV with a MegaView G2 CCD-camera (EMSIS, Muenster, Germany). Measurements of phage dimensions were calculated using samples that were stained with uranyl acetate (head sizes) or with phosphotungstic acid (all other dimensions).

### 4.6. DNA Isolation, Restriction and Sequencing

DNA extraction was performed as previously described [60]. CsCl purified phage particles were treated with DNase and RNase, followed by treatment with 10% SDS and proteinase K followed by DNA extraction with phenol: chloroform: isoamyl alcohol (25:24:1 *v*/*v*) and chloroform: isoamyl alcohol (24:1 *v*/*v*). DNA samples were digested with BamHI and ClaI, according to manufacturer’s protocols (New England BioLabs, Ipswich, MA, USA). The digested DNA was analyzed by agarose gel electrophoresis.

Prior to sequencing, DNA quality and quantity were estimated using both a Nanodrop (ND-1000, Thermo Fisher, Waltham, MA, USA) and by visualization after agarose gel electrophoresis. Genomic sequencing was outsourced to the Centre for Genomic Research at the University of Liverpool, Liverpool, UK. Illumina MiSeq system (Illumina, San Diego, CA, USA), with a TruSeq DNA Nano LT library sample preparation kit for library preparation. Library quality was assessed using the Agilent Bioanalyzer (Agilent Technologies, Santa Clara, CA, USA) and Qubit measurements prior to being sequenced with paired-end reads of 2 × 250 bp. Reads were assembled using Spades genome assembler v.3.10 (St. Petersburg, Russia).

### 4.7. Bioinformatic Analysis

ORFs of CB1, CB3, and CB4 were predicted with GLIMMER [61] and GenmarkS [62]. Further analysis of predicted ORF gene products was conducted with BLASTP (http://blast.ncbi.nlm.nih.gov/Blast.cgi?PAGE=Proteins), Pfam (http://pfam.xfam.org/search#tabview=tab1; [63]), InterProScan (https://www.ncbi.nlm.nih.gov/pmc/articles/PMC3998142/; [64]), and HHpred (https://toolkit.tuebingen.mpg.de/#/tools/hhpred; [65]). With the detection of ORFs with transmembrane domains and lipoprotein cleavage signal being identified with the use of TMHMM v.2 (http://www.cbs.dtu.dk/services/TMHMM/; [66]) and LipoP v.1 (http://www.cbs.dtu.dk/services/LipoP/; [67]), respectively. The molecular weights of the predicted ORFs were estimated using the batch protein molecular weight determination of the sequence manipulation suite (http://www.bioinformatics.org/sms2/protein_mw.html). The presence of transfer RNA genes was investigated with the use of tRNAscan-SE (http://lowelab.ucsc.edu/tRNAscan-SE/; [68]) and ARAGORN (http://130.235.46.10/ARAGORN/; [69]). Potential Rho-independent terminators in were identified using ARNold (http://rna.igmors.u-psud.fr/toolbox/arnold; [70]) with Mfold QuikFold (http://unafold.rna.albany.edu/?q=DINAMelt/Quickfold; [71]) using RNA energy rules 3.0 to verify predictions, with putative single-stranded hairpin promoters identification being assisted with Mfold QuikFold using DNA energy rules.

### 4.8. Comparative Genomics

The linear genomic comparison maps of CB1 and other N4-like phages were created with the use of either BLASTN or TBLASTX to determine genome homology and it was visualized with Easyfig [72]. Artemis Comparison Tool (ACT) was used for the identification of feature variations between the genomes of the CB1-like phages, with homology being assessed with BLASTN [73]. Phylogenetic analysis employed the use of the DNA polymerase and vRNA polymerase proteins of 38 N4-like phages as well as those of CB1-like phages (Supplementary Information 6, Table S2) using MEGA7 [74], involving the use of MUSCLE for sequence alignment [75], with the construction of phylograms using the maximum likelihood (ML) method that was based on the Jones–Thornton–Taylor model [76], with the robustness of the trees being assessed with bootstrapping (1000). The heat map comparing the genomes of 38 N4-like phages and CB1-like phages was generated using Gegenees, using accurate parameters (fragment length: 200 bp; step size: 100 bp; threshold: 0%) [77].

### 4.9. Phage CB1 Virion ESI-MS/MS Proteome Analysis

Phage capsid proteins were extracted from high titer CsCl purified phage (>1 × 10^9^ PFU/mL) using chloroform:methanol extraction (1:1:0.75, *v*/*v*/*v*). The resulting protein pellet was resuspended in loading buffer (1% SDS, 6% sucrose, 100 mM dithiothreitol, 10 mM Tris pH 6.8, 0.0625% *w*/*v* bromophenol blue) and heated to 95 °C for 5 min to resuspend the pellet. This was subsequently loaded onto a 12% SDS-PAGE gel, after which gel electrophoresis was conducted. The resulting gel was then stained using Gelcode™ Blue Safe Protein Stain (Thermo Fisher) to visualize virion proteins. Gel fragments were extracted and subjected to tyrosination, which were analyzed using ESI-MS/MS exactly, as described previously [78].

### 4.10. Biocontrol Assays to Determine Biocontrol Potential CB1, CB3 and CB4 Mixture

The potato whole tuber rot assay was used to assess the potential of a phage mixture comprised of phages CB1, CB3, and CB4 to prevent infection of potato tissue by *P. atrosepticum* and was conducted in a similar manner, as previously described [15]. The phage mixture was made by adding an equal number of phages to a total titer approx. 1 × 10^7^ PFU/mL to SM buffer. *P. atrosepticum* strains, DSM 18077 and DSM 30186, were resuspended in deionized water with cell numbers of approx. 1 × 10^7^ CFU/mL and mixed in equal quantities. Ware potato tubers were obtained at a local supermarket, these were first washed with tap water, then surface sterilized for 10 min in 70% isopropanol, then again washed with tap water, and allowed to dry on tissue paper at room temperature.

Whole tuber assay involved tubers being incised at the rose end (opposite the stolon end) to remove a 0.5 cm transverse slice. To which, 100 µL of either bacteria or water was added, left to absorb and then followed with the addition of 100 µL of SM buffer or phage mixture. These were then allowed 30 min to sit at room temperature before the detached potato slices were reattached using sterile toothpicks to tubers. Tubers were then incubated or 72 h at 25 °C in a humid box. To assess the protective effect of the phage mixture, the weight of rotten tissue was determined for each tuber.

### 4.11. Statistical Analyses of Data

Figure 7 was generated with Excel. Statistical analysis was preformed with IBM SPSS Software v. 24, (Armonk, NY, USA). Normality of data was assessed with the Shapiro-Wilks test at a significance level of 0.05. Non-parametric tests were chosen for data not normally distributed. Comparison of weight of soft rot tissue of infected potatoes, phage treated and untreated, were performed with the Mann-Withney U test.

### 4.12. Accession Number

The genome sequences of phages CB1, CB3, CB4 were submitted to GenBank under accession numbers KY514264, KY514265, and KY549659, respectively.

## Figures and Tables

**Table 1:** Host range of *Pectobacterium* phages CB1, CB3, and CB4 on 31 strains of various members of the soft rot *Enterobacteriaceae*, as determined by spot testing with serial dilutions of phage suspensions. The efficiency of plating (EOP) values were determined for sensitive strains.

Bacteria		Bacteriophage Infection		
Species	Strain	CB1	CB3	CB4
*Pectobacterium atrosepticum*	DSMZ 18077 (type strain)	1.000 *	2.263	0.909
	DSMZ 30184	2.00 × 10^−5^	CS	CS
	DSMZ 30185	0.371	0.126	0.136
	DSMZ 30186	0.003	1.000 *	1.000 *
	CB BL1-1	CS	CS	CS
	CB BL2-1	0.571	0.737	0.336
	CB BL3-1	0.529	0.605	0.218
	CB BL4-1	0.571	0.789	0.245
	CB BL5-1	CS	0.158	CS
	CB BL7-1	CS	CS	CS
	CB BL9-1	0.027	0.007	0.164
	CB BL11-1	CS	0.279	0.127
	CB BL12-2	2.86 × 10^−6^	0.079	0.255
	CB BL13-1	1.71 × 10^−5^	0.063	0.436
	CB BL14-1	CS	CS	CS
	CB BL15-1	-	-	-
	CB BL16-1	0.005	CS	CS
	CB BL18-1	CS	0.037	CS
	CB BL19-1	0.005	CS	CS
*Pectobacterium carotovorum* subsp. *carotovorum*	DSMZ 30168 (type strain)	-	-	-
	DSMZ 30169	-	-	-
	DSMZ 30170	-	-	-
	CB BL19-1-37	-	-	-
*Dickeya chrysanthemi* bv. *chrysanthemi*	LMG 2804	-	-	-
*Dickeya dianthicola*	PD 482	-	-	-
	PD 2174	-	-	-
	GBBC 1538	-	-	-
*Dickeya solani*	sp. PRI 2222	-	-	-
	LMG 25865	-	-	-
	GBBC 1502	-	-	-
	GBBC 1586	-	-	-

Results recorded as −, no infection; CS, presence of clear spot with no plaque formation; number, EOP value; * host strain of phage. EOP values determined by spot testing in triplicate.

**Table 2:** Dimensions of *Pectobacterium* phages CB1, CB3, and CB4 negatively stained with 2% (*w*/*v*) uranyl acetate (head sizes) or with 1% phosphotungstic acid (all other measurements).

Phage	Head (nm)	Tail Length (nm)	Collar Width (nm)	Whisker Length * (nm)	Whisker Ball Length (nm)	Whisker Ball Width (nm)
CB1	67.8 ± 4.4 (*n* = *19*)	23.9 ± 1.7 (*n = 8*)	18.5 ± 1.3 *(n = 8)*	23.4 ± 3.0 *(n = 14)*	12.3 ± 1.4 *(n = 16)*	6.3 ± 0.7 *(n = 17)*
CB3	71.8 ± 1.7 *(n = 6)*	25.8 ± 2.3 *(n = 8)*	19.6 ± 0.8 *(n = 7)*	25.6 ± 1.7 *(n = 7)*	12.0 ± 1.5 *(n = 9)*	7.4 ± 0.9 *(n = 9)*
CB4	70.4 ± 2.5 *(n = 12)*	25.5 ± 1.0 *(n = 5)*	19.3 ± 0.8 *(n = 6)*	23.5 ± 1.6 *(n = 3)*	11.8 ± 0.9 *(n = 5)*	6.6 ± 0.6 *(n = 5)*

* Whisker length from collar to distal end of the whisker ball.

**Table 3:** Results of tandem mass spectrometry of proteins of the *Pectobacterium* phage CB1 virion.

ORF	Predicted Function	Molecular Mass (kDa)	No. of Unique Peptides	Sequence Coverage %
CB1_57	Putative tail protein	15.59	6	71
CB1_61	Putative tail spike protein	104.98	19	29
CB1_62	Putative tail protein	26.01	10	55
CB1_77	Virion associated RNA polymerase (N4 gp50-like)	399.92	37	13
CB1_78	Unknown structural protein	41.64	15	52
CB1_79	Structural protein (N4 gp52-like)	14.2	3	19
CB1_81	Structural protein (N4 gp54-like)	28.82	9	53
CB1_83	Major capsid protein (N4 gp56-like)	43.33	33	75
CB1_86	Portal protein (N4 gp59-like)	83.31	27	45
CB1_90	Structural protein (N4 gp67-like)	33.72	9	40

## Supplementary Materials

The following are available online at http://www.mdpi.com/1424-8247/11/2/45/s1, Supplementary information 1 Figure S1: One-step-growth growth analysis of phage CB1 infection of *P. atrosepticum* strain DSM18077, phage CB3 infection of *P. atrosepticum* strain DSM30186 and phage CB4 infection of *P. atrosepticum* strain DSM30186, Supplementary information 1 Figure S2: Stability of *Pectobacterium* phages CB1, CB3 and CB4 to various pH values upon 24 h of exposure, Supplementary information 1 Figure S3. Stability of *Pectobacterium* phages CB1, CB3 and CB4 to various temperatures upon one-hour exposure, Supplementary information 1 Figure S4: Genomic DNA of *Pectobacterium* phage CB1, which had been digested with restriction enzyme ClaI, Supplementary information 1 Figure S5: Genomic DNA of *Pectobacterium* phage CB3, which had been digested with restriction enzyme ClaI, Supplementary information 1 Figure S6: Genomic DNA of *Pectobacterium* phage CB4, which had been digested with restriction enzyme ClaI, Supplementary information 1 Figure S7: Pictures of the typical observed outcomes for the tuber rot assays, Supplementary information 1 Table S1. Results of physiological, biochemical, *Pectobacterium* genus specific and *Pectobacterium atrosepticum* and *Pectobacterium carotovorum* subsp. *carotovorum* species specific PCRs and MALDI-TOF mass spectrometry on isolates obtained from potato stem samples symptomatic for blackleg from farms in Co. Cork, Ireland, Supplementary information 1, Table S2. Identified ORFs and tRNA gene variations between the genomes of *Pectobacterium* phages CB1, CB3 and CB4, Supplementary information 1, Table S3. Homologs of the eighteen core proteins described by Li et al. 2016 found present in the genomes of *Pectobacterium* phages CB1, CB3 and CB4, Supplementary information 1, Table S4. Putative single-stranded hairpin promoters predicted in the genomes of *Pectobacterium* phages CB1, CB3 and CB4 identified assisted with QuikFold, Supplementary information 1, Table S5. High ΔG rho-independent terminators predicted in the genome *Pectobacterium* phage CB1 identified using ARNold and QuikFold, Supplementary information 1, Table S6. High ΔG rho-independent terminators predicted in the genome *Pectobacterium* phage CB3 identified using ARNold and QuikFold, Supplementary information 1, Table S7. High ΔG rho-independent terminators predicted in the genome Pectobacterium phage CB4 identified using ARNold and QuikFold. Supplementary information 1, Table S8. Shared high ΔG putative rho-independent terminators among *Pectobacterium* phages CB1, CB3 and CB4. Supplementary information 2, Table S1. Genome annotation of *Pectobacterium* phage CB1, Supplementary information 3, Table S1. Genome annotation of *Pectobacterium* phage CB3, Supplementary information 4, Table S1. Genome annotation of *Pectobacterium* phage CB4, Supplementary information 5, Table S1. Output from ESI-MS/MS from analysis of *Pectobacterium* phage CB1 virion proteins, Supplementary information 6, Table S1. Bacteria strains used in the Isolation and the testing of host range of *Pectobacterium* phages CB1, CB3 and CB4, Supplementary information 6, Table S2. Table S2. Genbank details of N4-like phages used in phylograms and Gegenees analysis.
