# Supplementary material for: Novel N4-Like Bacteriophages of Pectobacterium atrosepticum
Source: Pharmaceuticals (Basel). 2018 May 14;11(2):45. doi: 10.3390/ph11020045 (PMC6027278; doi:10.3390/ph11020045)
Supplement: Supplementary file 1 [file pharmaceuticals-11-00045-s001.zip › Buttimer et al. pharmaceuticals-286782 Supp info 6.pdf]

Table S1. Bacteria strains used in the Isolation and the testing of host range of *Pectobacterium* phages CB1, CB3 and CB4.

Bacteria	strain	Isolation source
Pectobacterium atrosepticum	DSMZ 18077 (type strain)	Potato (*Solanum tuberosum*)
	DSMZ 30184	Potato (*Solanum tuberosum* cv. Bodenkraft)
	DSMZ 30185	Potato (*Solanum tuberosum*)
	DSMZ 30186	Potato (*Solanum tuberosum* cv. Maritta)
Pectobacterium carotovorum subsp. carotovorum	DSMZ 30168 (type strain)	Potato (*Solanum tuberosum*)
	DSMZ 30169	*Brassica oleracea* var. *capitata*
	DSMZ 30170	Potato (*Solanum tuberosum* "Maritta")
Dickeya chrysanthemi bv chrysanthemi	LMG 2804 (type strain)	*Chrysanthemum*
Dickeya dianthicola	PD 482	*Solanum tuberosum* cv. Ostara
	PD 2174	-
	GBBC 1538	-
Dickeya solani	sp. PRI 2222	-
	LMG 25865	*Solanum tuberosum* cv. Première
	GBBC 1502	-
	GBBC 1586	-

Table S2. Genbank details of N4-like phages used in phylograms and Gegenees analysis.

Phage	Genbank accession number	DNA polymerase	virion RNA polymerase
Achromobacter phage JWAlpha	KF787095.1	KF787095.1	YP_009004769.1
Achromobacter phage JWDelta	KF787094.1	KF787094.1	AHC56581.1
Achromobacter phage phiAxp-3	NC_028908.1	NC_028908.1	YP_009208706.1
Acinetobacter phage Presley	KF669658.1	KF669658.1	YP_009007647.1
Dinoroseobacter phage DFL12phi1	KJ621082.2	KJ621082.2	YP_009043702.1
Dinoroseobacter phage vBDshPR2C	KJ803031.1	KJ803031.1	AID16877.1
Enterobacter phage EcP1	NC_019485.1	NC_019485.1	YP_007003173.1
Erwinia phage Ea9-2	NC_023579.1	YP_009007430	YP_009007447.1
Erwinia phage vB_EamP_Gutmeister	KX098391.1	ANJ65360.1	ANJ65375.1
Erwinia phage vB_EamP_Rexella	KX098390.1	ANJ65282.1	ANJ65299.1
Erwinia phage vB_EamP-S6	NC_019514.1	NC_019514.1	YP_007005815.1
Escherichia phage ECBP1	JX415535.1	YP_006908814.1	YP_006908827.1
Escherichia phage N4	NC_008720.1	YP_950517.1	YP_950528.1
Escherichia phage Pollock	NC_027381.1	NC_027381.1	YP_009152160.1
Escherichia phage vB_EcoP_G7C	HQ259105.1	YP_004782168.1	YP_004782180.1
Escherichia phage vB_EcoP_PhAPEC7	KF562340.1	YP_009056171.1	YP_009056186.1
Pseudoalteromonas phage pYD6-A	JF974296.1	JF974296.1	YP_007674286.1
Pseudomonas phage inbricus	MG018928.1	ATW58103.1	ATW58114.1
Pseudomonas phage KPP21	LC064302.1	LC064302.1	YP_009218950.1
Pseudomonas phage LIT1	FN422399.1	YP_003358435.1	YP_003358468.1
Pseudomonas phage LUZ7	FN422398.1	FN422398.1	YP_003358355.1
Pseudomonas phage RWG	KM411958.1	AIZ94788.1	AIZ94822.1
Pseudomonas phage vB_Pae575P-3	KX171209.1	ANT44317.1	ANT44349.1
Pseudomonas phage ZC03	KU356690.1	KU356690.1	AMD43402.1
Pseudomonas phage ZC08	KU356691.1	KU356691.1	AMD43541.1
Roseophage DSS3P2	FJ591093.1	FJ591093.1	YP_002899070.1
Roseophage EE36P1	FJ591094.1	FJ591094.1	YP_002898988.1
Salmonella phage FSL SP-058	NC_021772.1	NC_021772.1	YP_008239463.1
Salmonella phage FSL SP-076	KC139520.1	KC139520.1	YP_008240191.1
Sulfitobacter phage phiCB2047-B	NC_020862	NC_020862	YP_007675808.1
Vibrio phage JA-1	KC438282.1	KC438282.1	YP_008126816.1
Vibrio phage JSF3	KY065148.1	APD18062.1	APD18049.1
Vibrio phage phi 1	KP280062.1	KP280062.1	YP_009198592.1
Vibrio phage pVa5	KX889068.1	KX889068.1	APC46019.1
Vibrio phage pVco-5	KY612839.1	ARM71084.1	ARM71100.1
Vibrio phage VBP32	HQ634196.1	HQ634196.1	YP_007676574.1
Vibrio phage VBP47	HQ634194.1	HQ634194.1	YP_007674140.1
Vibrio phage VCO139	KC438283.1	KC438283.1	AGI61882.1
