# Supplementary material for: Novel N4-Like Bacteriophages of Pectobacterium atrosepticum
Source: Pharmaceuticals (Basel). 2018 May 14;11(2):45. doi: 10.3390/ph11020045 (PMC6027278; doi:10.3390/ph11020045)
Supplement: Supplementary file 1 [file pharmaceuticals-11-00045-s001.zip › Buttimer et al. pharmaceuticals-286782 Supp info 1.pdf]

Table S1. Results of physiological, biochemical, *Pectobacterium* genus (*pel* gene) specific and *Pectobacterium atrosepticum* and *Pectobacterium carotovorum* subsp. *carotovorum* species specific PCRs and MALDI-TOF mass spectrometry on isolates obtained from potato stem samples symptomatic for blackleg from farms in Co. Cork, Ireland.

Isolate	Isolation source	Cavity formation on CVP medium (25 ºC, 48 hrs)	Growth on NA at 37ºC, 48 hrs	Production of reducing substance from sucrose	Acid production of reducing substance from sucrose	PCR - *Pel* gene (Darasse et al 1994)	PCR - *P. atrosepticum* (De Boer & Ward 1995)	PCR - *P. carotovorum* subsp. *carotovorum* (Kang et al 2003)	MALD-TOF MS	Identity
CB BL1-1	*Solanum tuberosum* cv. British Queen	+	-	+	+	+	+	NA	Pa	Pa
CB BL2-1	Solanum tuberosum cv. British Queen	+	-	+	+	+	+	NA	Pa	Pa
CB BL3-1	*Solanum tuberosum* cv. British Queen	+	-	+	+	+	+	NA	Pa	Pa
CB BL4-1	*Solanum tuberosum* cv. British Queen	+	-	+	+	+	+	NA	Pa	Pa
CB BL5-1	Solanum tuberosum cv. British Queen	+	-	+	+	+	+	NA	Pa	Pa
CB BL7-1	Solanum tuberosum cv. Golden wonder	+	-	+	+	+	+	NA	Pa	Pa
CB BL9-1	Solanum tuberosum cv. Golden wonder	+	-	+	+	+	+	NA	Pa	Pa
CB BL11-1	*Solanum tuberosum cv.* Rooster	+	-	+	+	+	+	NA	Pa	Pa
CB BL12-2	*Solanum tuberosum* cv. Golden wonder	+	-	+	+	+	+	NA	Pa	Pa
CB BL13-1	*Solanum tuberosum* cv. Golden wonder	+	-	+	+	+	+	NA	Pa	Pa
CB BL14-1	*Solanum tuberosum* cv. Golden wonder	+	-	+	+	+	+	NA	Pa	Pa
CB BL15-1	*Solanum tuberosum* cv. Golden wonder	+	-	+	+	+	+	NA	Pa	Pa
CB BL16-1	*Solanum tuberosum* cv. Golden wonder	+	-	+	+	+	+	NA	Pa	Pa
CB BL18-1	*Solanum tuberosum* cv. Golden wonder	+	-	+	+	+	+	NA	Pa	Pa
CB BL19-1	*Solanum tuberosum* cv. Golden wonder	+	-	+	+	+	+	NA	Pa	Pa
CB BL19-1-37	*Solanum tuberosum* cv. Golden wonder	+	+	-	-	+	-	+	NA	Pcc

Results recorded as +, positive; −, negative; NA, not available; Pa, *P. atrosepticum*; Pcc, *P. carotovorum* subsp. *carotovorum*

(A)

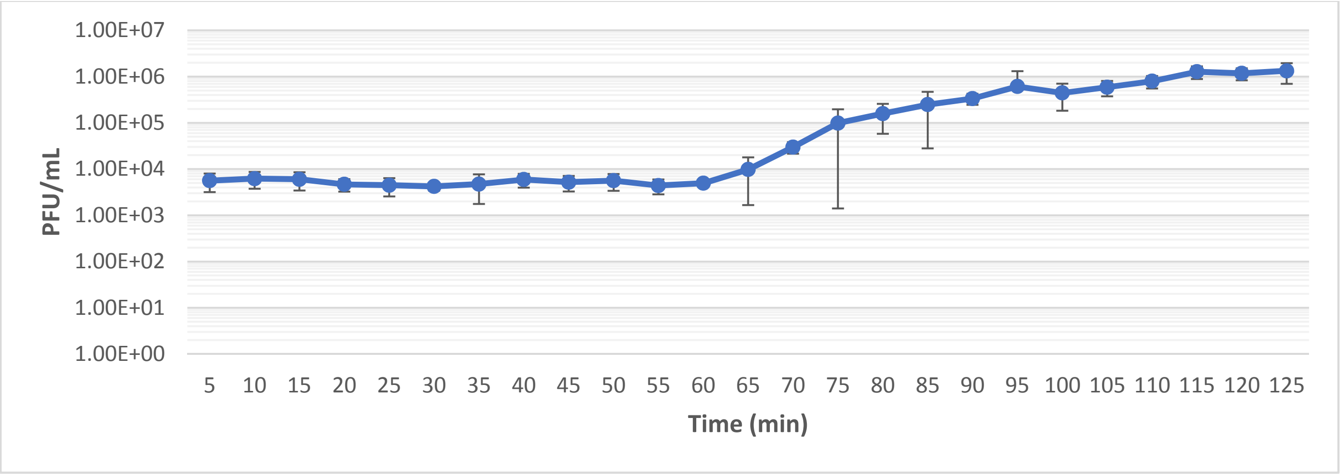
1.00E+07
1.00E+06
1.00E+05
1.00E+04
1.00E+03
1.00E+02
1.00E+01
1.00E+00
PFU/mL
5 10 15 20 25 30 35 40 45 50 55 60 65 70 75 80 85 90 95 100 105 110 115 120 125
Time (min)

(B)

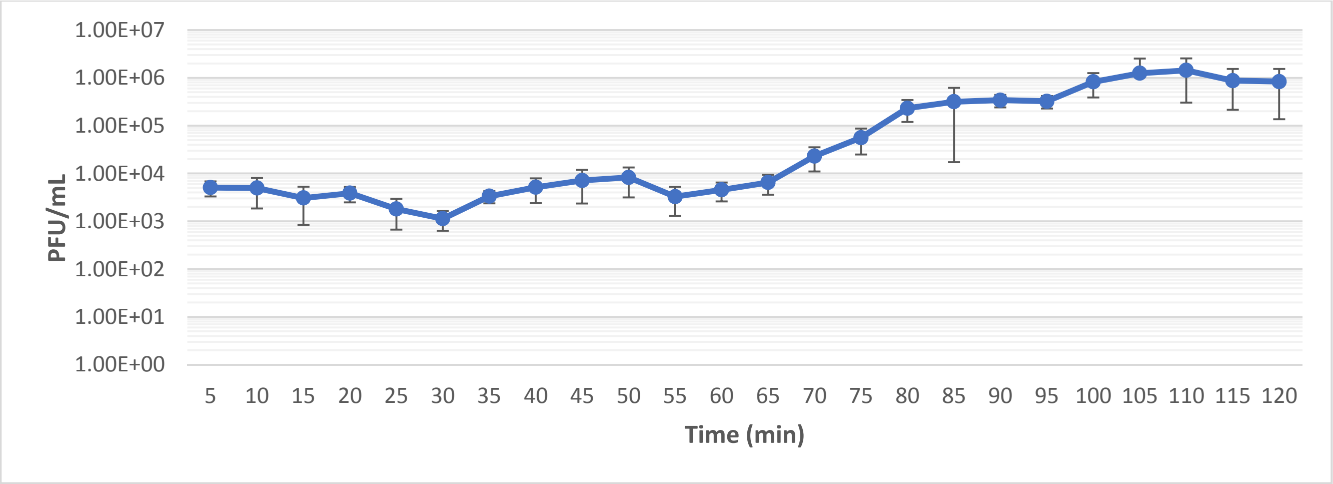
1.00E+07
1.00E+06
1.00E+05
1.00E+04
1.00E+03
1.00E+02
1.00E+01
1.00E+00
PFU/mL
5 10 15 20 25 30 35 40 45 50 55 60 65 70 75 80 85 90 95 100 105 110 115 120
Time (min)

(C)

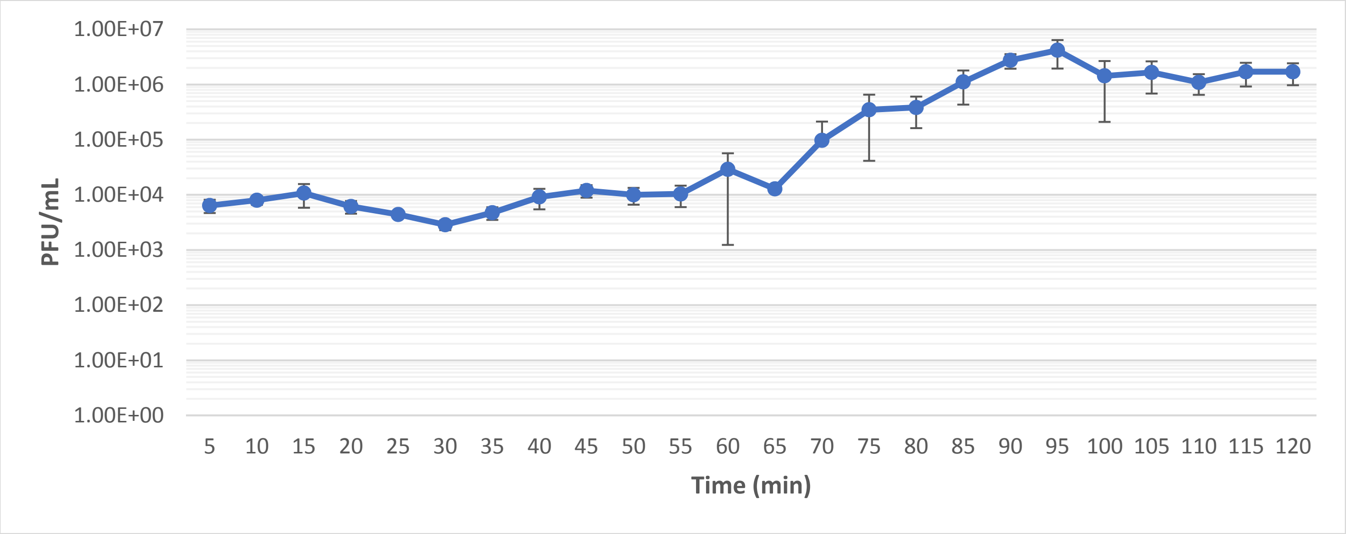

Figure S1. Single step growth curve growth analysis of phage CB1 infection of *P. atrosepticum* strain DSM18077 (A), phage CB3 infection of *P. atrosepticum* strain DSM30186 (B) and phage CB4 infection of *P. atrosepticum* DSM30186 (C). Each assay was independently repeated in triplicate and the results were averaged.

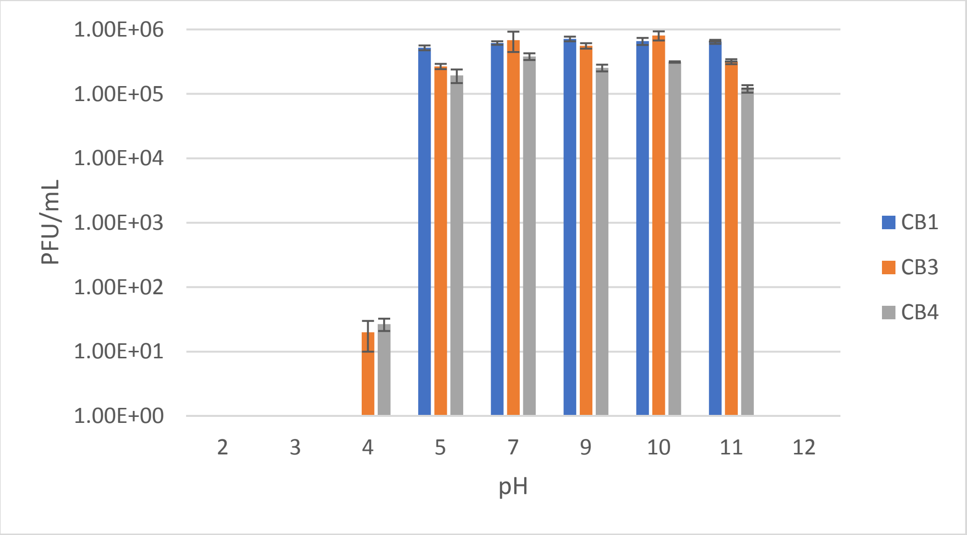

Figure S2. Stability of *Pectobacterium* phages CB1, CB3 and CB4 to various pH values upon 24 hours of exposure. Error bars represent standard deviation of biological repeats (n=3).

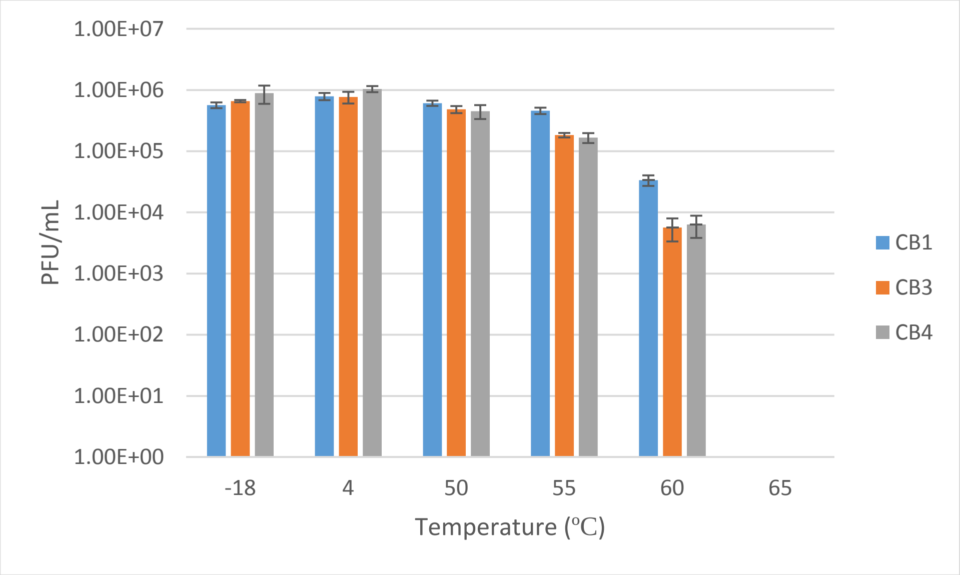

Figure S3. Stability of *Pectobacterium* phages CB1, CB3 and CB4 to various temperatures upon one-hour exposures. Error bars represent standard deviation of biological repeats (n=3).

Table S2. Identified ORFs and tRNA gene variations between the genomes of *Pectobacterium* phages CB1, CB3 and CB4. With genome comparisons made using BLASTN with ACTs. Colour coded: Green (shared feature), Yellow (unique feature to phage in question).

CB1	CB3	CB4	Variation discription
CB1_4 (hypothetical)	_	_	CB1_4 has no homolog in CB3 + CB4
CB1_8 (hypothetical)	_	_	CB1_8 has no homolog in CB3 + CB4
CB1_10 (hypothetical)	CB3_9 (hypothetical)	CB4_9 (hypothetical)	ORFs of CB1 are not share with CB3 + CB4
CB1_11 (hypothetical)	CB3_10 (hypothetical)	CB4_10 (hypothetical)	
_	CB3_11 (hypothetical)	CB4_11 (hypothetical)	
_	CB3_17 (hypothetical)	CB4_17 (hypothetical)	CB3/CB4_17 homolog not present in CB1
CB1_21 (hypothetical)	CB3_22 (hypothetical)	CB4_22 (hypothetical)	ORF of CB1 are not shared with CB3 + CB4
_	CB3_23 (hypothetical)	CB4_23 (hypothetical)	
CB1_24 (HNH)	CB3_26 (hypothetical)	CB4_26 (hypothetical)	ORFs of CB1 are not shared with CB3 + CB4
_	CB3_27 (hypothetical)	CB4_27 (hypothetical)	
CB1_26 (hypothetical)	CB3_29 (hypothetical)	CB4_29 (hypothetical)	ORF of CB1 different in part to CB3 + CB4
CB1_39 (HNH)	_		CB1 has no homolog in CB3 + CB4
CB1_42 (N4 gp32 like)	CB3_45 (N4 gp32 like)	CB4_44 (N4 gp32 like)	N4 gp32-like gene of CB3 different to CB1+CB4
CB1_45 (rIIB)	CB3_49 (rIIB)	ORF48 (rIIB)	Difference of CB1 ORF length compared to CB3 + CB4
CB1_61 (tail spike)	CB3_66 (tail spike)	CB4_64, 65 (tail spike)	CB4 homolog split into two ORFs
CB1_63 (thymidylate synthase)	CB3_68 (thymidylate synthase)	ORF67 (thymidylate synthase)	ORF of CB1 and CB3 different to CB4
_	_	2 tRNA genes (tRNA1 and tRNA2)	CB4 possess tRNA genes not present in CB1 + CB3

Table S3. Homologs of the eighteen core proteins described by Li et al. 2016 found present in the genomes of *Pectobacterium* phages CB1, CB3 and CB4.

No.	N4-like Core genes	CB1 homolog	CB3 homolog	CB4 homolog
1	RNA P1	CB1_22	CB3_24	CB4_24
2	RNA P2	CB1_23	CB3_25	CB4_25
3	gp24 N4	CB1_35	CB3_38	CB4_38
4	gp25 N4	CB1_38	CB3_41	CB4_41
5	DNA P/ gp39 N4	CB1_46	CB3_50	CB4_49
6	gp42 N4	CB1_66	CB3_71	CB4_70
7	DNA primase	CB1_67	CB3_72	CB4_71
8	gp44 N4	CB1_70	CB3_75	CB4_74
9	SSB/gp45 N4	CB1_72	CB3_77	CB4_76
10	vRNAP	CB1_77	CB3_82	CB4_81
11	gp53 N4	CB1_80	CB3_85	CB4_84
12	gp54 N4	CB1_81	CB3_86	CB4_85
13	gp55 N4	CB1_82	CB3_87	CB4_86
14	MCP/ gp56 N4	CB1_83	CB3_88	CB4_87
15	gp57 N4	CB1_84	CB3_89	CB4_88
16	94kDa protein/ gp59 N4	CB1_86	CB3_91	CB4_90
17	terminase A	CB1_91	CB3_97	CB4_95
18	gp69	CB1_93	CB3_99	CB4_97

Table S4. Putative single-stranded hairpin promoters predicted in the genomes of *Pectobacterium* phages CB1, CB3 and CB4 identified assisted with QuikFold.

**Phage**	**Promotor**	**Coordinates**	**Sequence**
CB1	Porf1_1	76 - 105 bp	GTGTTGAACCGGTATCCGGTACAGTACCGT
	Porf1_2	823 - 852 bp	ATGGCATCCATGCATCATGGCATCCATATG
	Porf1_5	2085 - 2114 bp	TTGGACCGAGCGTATCGCTCAGCCCACTTA
CB3	Porf3_1	130 - 159 bp	GTGTTGAACCGGTATCCGGTACAGTACCGT
	Porf3_ 2	919 - 948 bp	ATGGCATCCATGCATCATGGCATCCATATG
	Porf3_4	1850 - 1879 bp	TCGGACCGCACGTATCGTGCAGCCCACTTA
CB4	Porf4_1	76 - 105 bp	GTGTTGAACCGGTATCCGGTACAGTACCGT
	Porf4_2	866 - 895 bp	ATGGCATCCATGCATCATGGCATCCATATG
	Porf4_4	1797 - 1826 bp	TCGGACCGCACGTATCGTGCAGCCCACTTA

Table S5. High ΔG rho-independent terminators predicted in the genome *Pectobacterium* phage vB_PatP_CB1 identified using ARNold and QuikFold.

**Terminator**	**Coordinates**	**Sequence**	**ΔG kcal/mol**
Torf1_1	397 - 428	GCCTACTCTTCGGAGTAGGCTTATTCTTTTCT	-17.2
Torf1_7	2,906 - 2,935	TAACCCCTTCGGGGGTTATCTTATTTTTTA	-13.6
Torf1_20	7,127 - 7,158	GCCATCCCTTCGGGGATGGCTGTTTTATTGAG	-19.3
Torf1_26	11,034 - 11,070	CGGACTCCCTAAGATGGGGAGTCCGTATTTTTTCATA	-19
Torf1_45	25,131 - 25,163	GCCAGCCCTTCGGGGCTGGTTTTTTAATATCAT	-18.8
Torf1_47	28,088 - 28,113	GCCCCTTCGGGGGCTTTTTTGAGGCT	-13.7
Torf1_51	29,497 - 29,525	AGCCCCTAACGGGGCTTTTTTATTGAGGT	-12.8
Torf1_56	31,765 - 31,793	GCCCACCTAGTGTGGGCTTATATTAATCT	-10.7
Torf1_57	31,751 - 31,782, complement	AGCCCACACTAGGTGGGCTTTTTTATAGCATC	-11.8
Torf1_61	33,531 - 33,561, complement	GGGAGCCTAATGGCTCCCTTTTTAATCTGGA	-15.4
Torf1_72	45,051 - 45,078	GCCCACTTCGGTGGGCTTTTTTATCTAT	-14.7
Torf1_76	46,958 - 46,987	GCCCCTCGATTGAGGGGCTTTATTTTTTAG	-14.5
Torf1_77	46,944 - 46,976, complement	AGCCCCTCAATCGAGGGGCTTTTTATTACGGTA	-15.4
Torf1_81	62,438 - 62,467, complement	GGGAGCTTAAGGCTCCCTTTTCATTGTGAG	-11.9
Torf1_83	63,896 - 63,928, complement	GCCGGGGATAATTCCCCGGCTTTTTTATATCTA	-18.1
Torf1_87	69,766 - 69,796	CTCCCTCTTCAGAGGGAGcTTTTAAACCTGA	-12.1
Torf1_88	70,620 - 70,649	GCCCCACTTCGGTGGGGCTTTTTCCGTTAT	-18
Torf1_89	71,047 - 71,076	GCCCCCGATTAAGGGGGCTTTTTTATTACA	-14.6

Table S6. High ΔG rho-independent terminators predicted in the genome *Pectobacterium* phage vB_PatP_CB3 identified using ARNold and QuikFold.

**Terminator**	**Coordinates**	**Sequence**	**ΔG kcal/mol**
Torf3_1	451 - 482	GCCTACTCTTCGGAGTAGGCTTATTCTTTTCT	-17.2
Torf3_7	2,670 - 2,699	TAACCCCTTCGGGGGTTATCTTATTTTTTA	-13.6
Torf3_21	7,283 - 7,314	GCCATCCCTTCGGGGATGGCTGTTTTATTGAG	-19.3
Torf3_29	11,386 - 11,422	CGGACTCCCCATCTTAGGGAGTCCGTATTTTTTCAT A	-17.1
Torf3_57	20,329 - 20,357	GCCAGATTTAATCTGGCTTTTTCTTTTTA	-9.4
Torf3_49	25,440 - 25,472	GCCAGCCCTTCGGGGCTGGTTTTTTAATATCAT	-18.8
Torf3_51	28,397 - 28, 422	GCCCCTTCGGGGGCTTTTTTGAGGCT	-13.7
Torf3_55	29,806 - 29, 834	AGCCCCTAACGGGGCTTTTTTATTGAGGT	-12.8
Torf3_60	32,074 - 32,102	GCCCACCTAGTGTGGGCTTATATTAATCT	-10.7
Torf3_65	33,840 - 33,870, complement	GGGAGCCTAATGGCTCCCTTTTTAATCTGGA	-15.4
Torf3_77	45,360 - 45, 387	GCCCACTTCGGTGGGCTTTTTTATCTAT	-14.7
Torf3_81	47,267 - 47,296	GCCCCTCGATTGAGGGGCTTTATTTTTTAG	-14.5
Torf3_82	47, 253 - 47, 285, complement	AGCCCCTCAATCGAGGGGCTTTTTATTACGGTA	-15.4
Torf3_86	62,747 - 62,776, complement	GGGAGCTTAAGGCTCCCTTTTCATTGTGAG	-11.9
Torf3_88	64,205 - 64,237, complement	GCCGGGGATAATTCCCCGGCTTTTTTATATCTA	-18.4
Torf3_92	69,948 - 69,978	CTCCCTCTTCAGAGGGAGcTTTTAAACCTGA	-12.1
Torf3_94	70,802 - 70,831	GCCCCACTTCGGTGGGGCTTTTTCCGTTAT	-18
Torf3_95	71,229 - 71,258	GCCCCCGATTAAGGGGGCTTTTTTATTACA	-14.6

Table S7. High ΔG rho-independent terminators predicted in the genome *Pectobacterium* phage vB_PatP_CB4 identified using ARNold and QuikFold.

**Terminator**	**Coordinates**	**Sequence**	**ΔG kcal/mol**
Torf4_1	396 - 429	GCCTCATCCTTCGGGATGGGGCTATCTCTTTTCT	-19.6
Torf4_7	2,617 - 2,646	TAACCCCTTCGGGGGTTATCTTATTTTTTA	-13.6
Torf4_21	7,230 - 7,261	GCCATCCCTTCGGGGATGGCTGTTTTATTGAG	-19.3
Torf4_29	11,333 - 11,369	CGGACTCCCCATCTTAGGGAGTCCGTATTTTTTCAT A	-17.1
Torf4_45	20,116 - 20,144	GCCAGATTTAATCTGGCTTTTCTTTTTAA	-9.4
Torf4_48	25,226 - 25,258	GCCAGCCCTTCGGGGCTGGTTTTTTAATATCAT	-18.8
Torf4_50	28,183 - 28,208	GCCCCTTCGGGGGCTTTTTTGAGGCT	-13.7
Torf4_54	29,592 - 29,620	AGCCCCTAACGGGGCTTTTTTATTGAGGT	-12.8
Torf4_59	31,860 - 31,888	GCCCACCTAGTGTGGGCTTATATTAATCT	-10.7
Torf4_64	33,826 - 33,656, complement	GGGAGCCTAATGGCTCCCTTTTTAATCTGGA	-15.4
Torf4_76	45,615 - 45,642	GCCCACTTCGGTGGGCTTTTTTATCTAT	-14.7
Torf4_81	47,508 - 47,540, complement	AGCCCCTCAATCGAGGGGCTTTTTATTACGGTA	-15.4
Torf4_80	47,522 - 47,551	GCCCCTCGATTGAGGGGCTTTATTTTTTAG	-14.5
Torf4_85	63,002 - 63,031, complement	GGGAGCTTAAGGCTCCCTTTTCATTGTGAG	-18.1
Torf4_91	70,203 - 70,233	CTCCCTCTTCGGAGGGAGcTTTTAAACCTGA	-15.2
Torf4_92	71,175 - 71,204	GCCCCACTTCGGTGGGGCTTTTTCCGTTAT	-18
Torf4_93	71,602 - 71,631	GCCCCCGATTAAGGGGGCTTTTTTATTACA	-14.6

Table S8. Shared high ΔG putative rho-independent terminators among *Pectobacterium* phages CB1, CB3 and CB4

no.	CB1	CB3	CB4
1	Torf1_01	Torf3_01	Torf4_01
2	Torf1_07	Torf3_07	Torf4_07
3	Torf1_20	Torf3_21	Torf4_21
4	Torf1_26	Torf3_29	Torf4_29
5	Torf1_45	Torf3_49	Torf4_48
6	Torf1_47	Torf3_51	Torf4_50
7	Torf1_51	Torf3_55	Torf4_54
8	Torf1_56	Torf3_60	Torf4_59
9	Torf1_61	Torf3_65	Torf4_64
10	Torf1_72	Torf3_77	Torf4_76
11	Torf1_76	Torf3_81	Torf4_80
12	Torf1_77	Torf3_82	Torf4_81
13	Torf1_81	Torf3_86	Torf4_85
14	Torf1_87	Torf3_92	Torf4_91
15	Torf1_88	Torf3_94	Torf4_92
16	Torf1_89	Torf3_95	Torf4_93

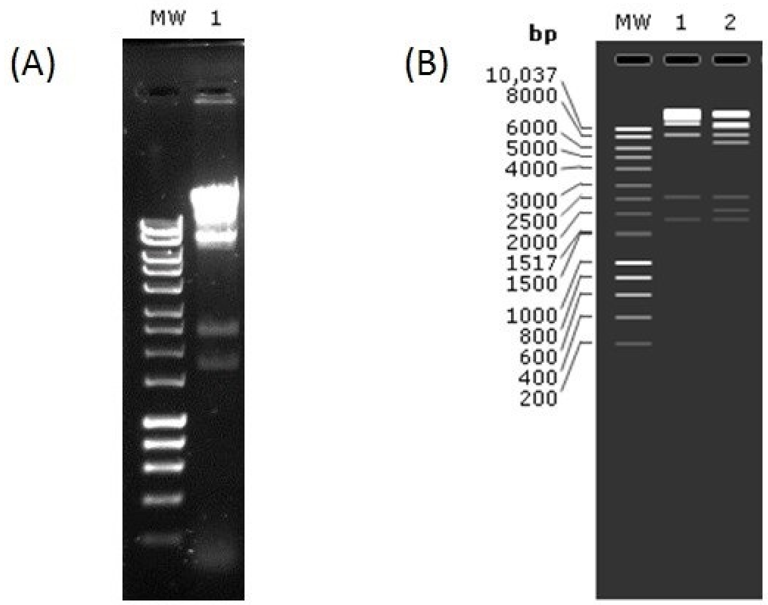

Figure S4. (A) Genomic DNA of *Pectobacterium* phage CB1, which had been digested with restriction enzyme ClaI (lane 1), with DNA marker (Hyperladder 1kb, Bioline) (lane MW). (B) *In silico* digest of CB1 redundant genomic DNA with ClaI with Dam methylation (lane 2); non-Dam methylation (lane 3); DNA marker (Hyperladder 1kb, Bioline) (lane MW). Gel concentration 1 % w/v agarose. Image B was generated using Snapgene.

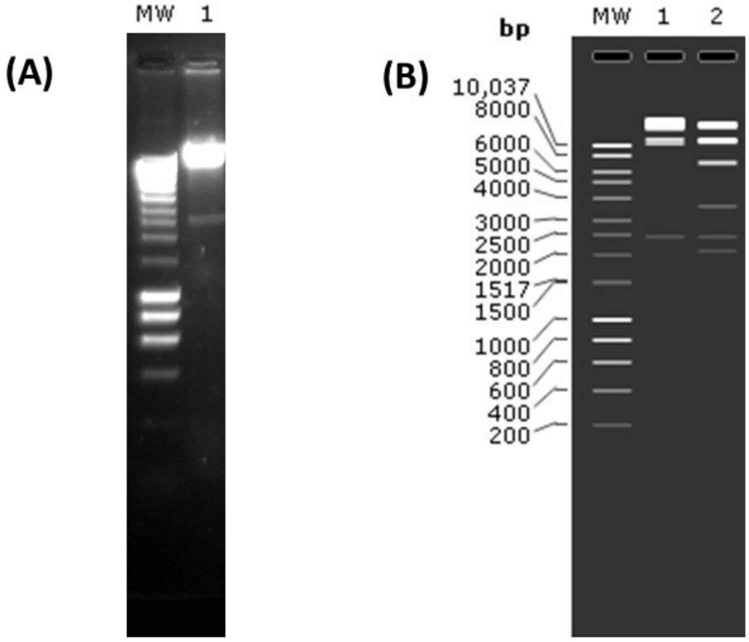

Figure S5. (A) Genomic DNA of *Pectobacterium* phage CB3, which had been digested with restriction enzyme ClaI (lane 1), with DNA marker (Hyperladder 1kb, Bioline) (lane MW). (B) *In silico* digest of CB3 redundant genomic DNA with ClaI with Dam methylation (lane 2); non-Dam methylation (lane 3); DNA marker (Hyperladder 1kb, Bioline) (lane MW). Gel concentration 1 % w/v agarose. Image B was generated using Snapgene.

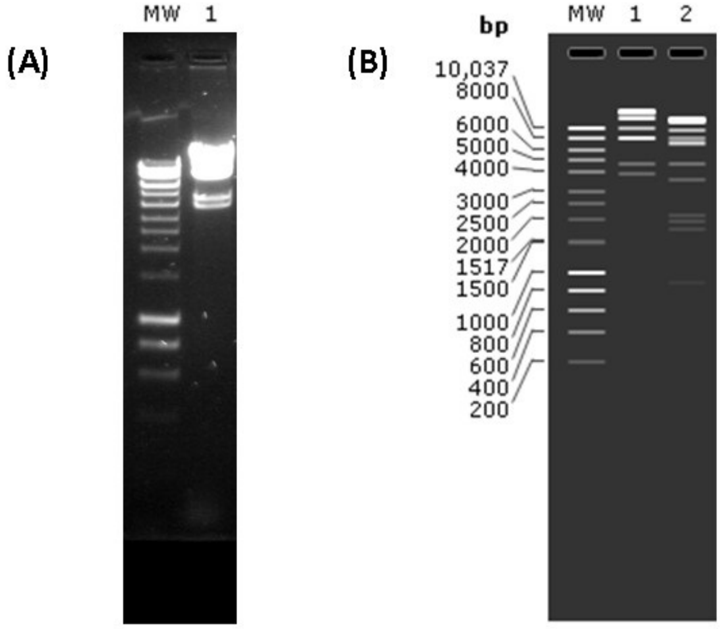

Figure S6. (A) Genomic DNA of *Pectobacterium* phage CB4, which had been digested with restriction enzyme ClaI (lane 1), with DNA marker (Hyperladder 1kb, Bioline) (lane MW). (B) *In silico* digest of CB4 redundant genomic DNA with ClaI with Dam methylation (lane 2); non-Dam methylation (lane 3); DNA marker (Hyperladder 1kb, Bioline) (lane MW). Gel concentration 1 % w/v agarose. Image B was generated using Snapgene.

(A) (B) (C)

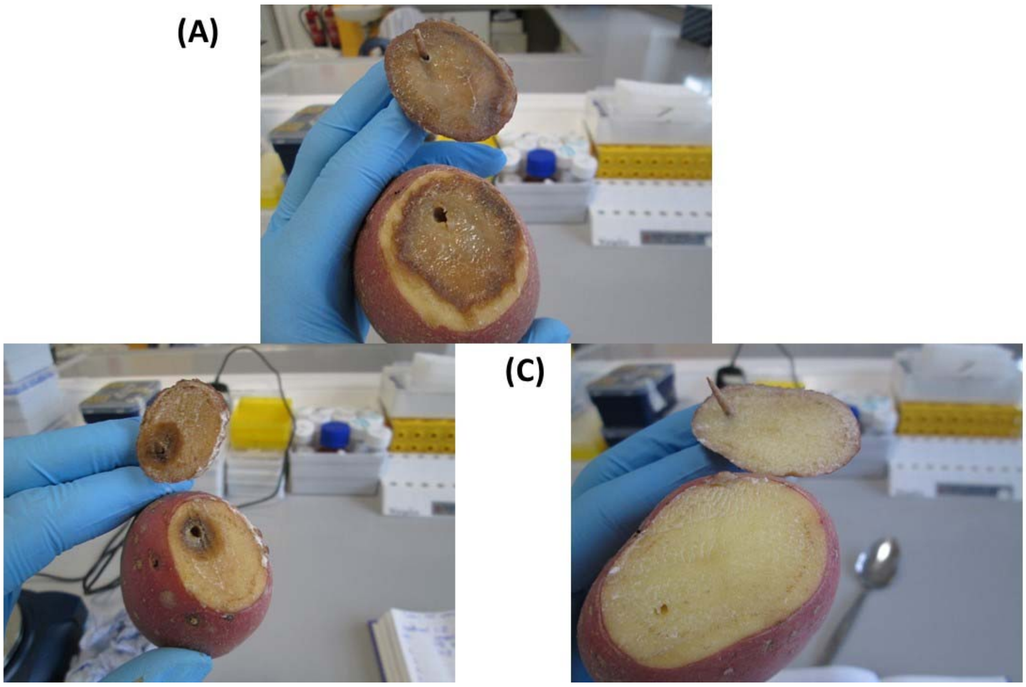

Figure S7. Pictures of the typical observed outcomes for the tuber rot assays. A: tuber treated with bacterial strains DSM 18077 + DSM 30186 and SM buffer. B: tuber treated with bacterial strains DSM 18077 + DSM 30186 and phage mixture (CB1 + CB3 + CB4). C: tuber treated with water and SM buffer.
